# Spatial host-microbiome profiling demonstrates bacterial-associated host transcriptional alterations in pediatric ileal Crohn’s disease

**DOI:** 10.1186/s40168-025-02178-8

**Published:** 2025-08-23

**Authors:** Sooyoung Jang, Eun Joo Lee, Sowon Park, Hyeji Lim, Byungsoo Ahn, Yoon Huh, Hong Koh, Yu Rang Park

**Affiliations:** 1 Department of Biomedical Systems Informatics, Yonsei University College of Medicine, Seoul, Republic of Korea; 2 Division of Gastroenterology, Hepatology and Nutrition, Department of Pediatrics, Yonsei University College of Medicine, Severance Fecal Microbiota Transplantation Center, Severance Hospital, Seoul, Republic of Korea; 3 Department of Pediatrics, University of Ulsan College of Medicine, Asan Medical Center Children’s Hospital, Seoul, Republic of Korea

**Keywords:** Gastrointestinal microbiome, Multi-omics, Host-microbiome interactions

## Abstract

**Background:**

Crohn’s disease (CD) is a chronic inflammatory bowel disease involving complex relationships between the gut microbiome and host immune system. However, the spatial relationships between tissue-resident bacteria and host cells in CD pathogenesis remain poorly understood. We developed a spatial host-microbiome profiling approach to simultaneously detect host transcriptomics and bacterial species at high taxonomic resolution in pediatric ileal CD tissues.

**Results:**

In this prospective case–control study, we analyzed 14 terminal ileal tissue samples from six pediatric patients with ileal CD and two controls. Spatial host-microbiome sequencing, combined spatial transcriptomics and in-situ polyadenylation, and bulk shotgun metagenome sequencing were performed. We developed a comprehensive bioinformatics pipeline to identify bacterial species and analyze host-microbiome interactions at cellular resolution, resulting in 13,876 analyzed cells. Our approach revealed increased bacterial abundance in CD tissues compared with controls. The extent of bacterial infiltration at diagnosis correlated with disease prognosis and severity of endoscopic findings. We identified 16 potentially beneficial and nine pathogenic microbiome members in ileal CD, including several newly discovered risk-modulating bacterial species. Cell-type-specific host gene expression analysis revealed transcriptome alterations related to bacterial defense mechanisms in the presence of various bacterial species.

**Conclusions:**

Our spatial host-microbiome profiling approach enables simultaneous species-level identification of bacteria and host transcriptomics. It reveals the intricate interactions between host cells and bacteria, providing cellular-level insights into CD pathogenesis. Our approach offers a powerful tool for investigating host-microbiome interactions in various microbiome-associated diseases to direct new strategies for microbiome-based therapeutics and prognostic markers.

Video Abstract

**Supplementary Information:**

The online version contains supplementary material available at 10.1186/s40168-025-02178-8.

## Background

The etiology of Crohn’s disease (CD) remains incompletely understood, although accumulating evidence has emphasized the potential importance of interactions between the gut microbiome and aberrant host immune responses in genetically predisposed patients [1–3]. Several studies have emphasized the importance of bacterial translocation in CD pathogenesis. Genome-wide association studies have identified CD-associated defects in microbe sensing, epithelial barrier function, microbicidal mechanisms, cytokine regulation, and adaptive immunity that potentially compromise host defense against bacterial translocation [4]. Furthermore, CD mouse models develop the disease in the presence of bacteria, but not in germ-free conditions [5, 6]. Recently, Ha et al. reported that a subset of mucosa-associated gut bacteria translocate into intestinal tissue in CD, potentially contributing to disease progression [7].

Consequently, numerous studies have attempted to characterize the gut microbiome composition in patients with CD [8–11]. They suggested that the gut microbiome in CD is characterized by an increase in pathogenic bacteria such as *Cutibacterium acnes* and *Haemophilus parainfluenzae*, and a decrease in beneficial bacteria like *Faecalibacterium prausnitzii* [9, 10, 12]. Understanding these microbial alterations is crucial for developing microbiome-based therapeutics to reduce disease incidence and severity [13, 14]. However, previous bulk metagenome studies on CD tissue microbiome have been limited by the use of homogenized tissue samples with fecal remnant, hindering the identification of spatial relationships between the bacteria and specific host cell types [8–11]. This limitation underscores the need for more advanced techniques to reveal the precise localization and interactions of microbes within the host intestinal tissues for understanding CD pathogenesis.

Recent advancements in spatial transcriptomics have revolutionized our understanding of cellular heterogeneity and spatial organization in complex biological systems [15]. However, previous methods relied on poly(A) tail-targeting approaches, limiting their application to eukaryotic mRNA, such as those of humans and mice [16]. Identifying non-host RNA, particularly bacterial RNA, which lacks poly(A) tails became challenging [17]. This hindered a comprehensive understanding of host-microbiome interactions and their potential roles in the pathogenesis of microbiome-associated diseases, such as CD. Nevertheless, Galeano et al. recently demonstrated that bacteria within tumors may play a crucial role in cancer metastasis using a spatial transcriptomics method targeting bacterial 16S rRNA [18]. However, this approach has limited taxonomic resolution when profiling the microbiome, restricting bacterial identification to the genus level, thereby hindering species-level discrimination [18–20].

To overcome these limitations and advance our understanding of CD pathogenesis, we developed a spatial host-microbiome profiling approach that allows for species-level identification of bacteria while simultaneously capturing host transcriptomics. Using this method, we examined bacterial localization patterns in pediatric CD tissues and their association with host transcriptome alterations at cellular resolution. Additionally, we identified and characterized microbiomes potentially associated with beneficial or pathogenic effects in pediatric CD. This approach provides insights into CD pathophysiology at the cellular level and offers potential applications for understanding host-microbiome interactions in various microbiome-associated diseases.

## Methods

### Participant and ethics

This prospective study included patients with CD from the Sinchon Severance Hospital, in Seoul. Ethical approval was obtained from the Institutional Review Board of Severance Hospital, Yonsei University College of Medicine (IRB number: 4–2022-1127). The study was conducted in adherence to the STROBE guidelines for observational studies (Additional file 1).

We recruited pediatric patients aged 7–18 years between November 01, 2022, and April 30, 2024. For the CD group, we included patients newly diagnosed with ileal CD. The control group consisted of patients with irritable bowel syndrome who underwent endoscopy but showed no evidence of inflammation on endoscopic examination, histopathological analysis of biopsy specimens, or blood tests (Supplementary Table S1). Both the patients and their legal guardians provided informed consent for participation in the study. Clinical relapse of pediatric CD was defined as a Pediatric Crohn’s Disease Activity Index score ≥ 30, indicating worsening of the disease [21].

### Sample collection and storage

Tissue samples were collected from the terminal ileum during endoscopic examination. For patients with CD, both inflamed and non-inflamed tissues were obtained, while control group samples consisted of only non-inflamed tissue. Immediately after collection, these tissue samples were embedded in optimum cutting temperature compound (SciGen Scientific, Gardena, CA, USA), frozen, and stored at − 80 °C. The stored samples were subsequently processed for spatial total RNA sequencing and bulk shotgun metagenome sequencing.

### Spatial total RNA sequencing

Tissue block quality was evaluated by measuring the RNA Integrity Number (RIN) using an Agilent 4200 TapeStation system (Agilent Technologies, Santa Clara, CA, USA). Samples with RIN ≥ 4 were included. Due to the small size of the tissues, multiple sections were placed on each sample block for analysis. In the CD group, both inflamed and non-inflamed tissue sections from the same patient were mounted side by side, while only non-inflamed tissue sections were mounted for the control group.

Following the approach by McKellar et al., we conducted in-situ polyadenylation [17]. This included equilibration with wash buffer containing Protector RNase Inhibitor (Roche, catalog no. 3335402001), followed by incubation with an enzyme mix containing yeast poly(A) polymerase (Thermo Scientific, catalog no. 74225Z25KU). After the in-situ polyadenylation step, we prepared final sequencing libraries using standard Visium library preparation protocol (CG000239, Rev F, 10 × Genomics). The prepared libraries were sequenced on a NovaSeq 6000 system (Illumina, San Diego, CA, USA), using the NovaSeq 6000 S1 Reagent Kit v1.5 (200 cycles, 20,028,318, Illumina), to a depth of approximately 120 M reads per sample.

### Bulk shotgun metagenome sequencing

Genomic DNA quality and quantity were assessed via fluorometry (Qubit, Invitrogen) and gel electrophoresis. Samples with a DNA Integrity Number ≥ 6, as measured by an Agilent 4200 TapeStation, were used. The library was prepared using the Illumina TruSeq Nano DNA Library Prep Kit. For each sample, 100 ng of DNA was fragmented to 350 bp using a Qsonica 800 R2, followed by Illumina adapter ligation and PCR amplification. Libraries sized 500–600 bp were selected and quantified using TapeStation 4200 (Agilent Technologies) and KAPA Library Quantification Kit (Kapa Biosystems). Sequencing was performed on an Illumina NovaSeq 6000 platform using 150 bp paired-end reads.

### Host transcriptome analysis

Host transcriptomics data were preprocessed using Space Ranger v1.3.1 (10 × Genomics). Downstream analyses with the output count matrices were performed using Scanpy v1.9.3 [22]. As the Visium method captures multiple cells within each spot, we employed Cell2location, a computational tool that maps single-cell RNA sequencing data onto spatial transcriptomics data [23]. For this process, we utilized previous single-cell RNA sequencing data from pediatric patients with CD [24]. Cell2location integrates this single-cell data with our spatial transcriptomics data, allowing us to identify areas enriched with specific cell types across the analyzed tissue samples. Low-quality spots with > 40% mitochondrial reads were excluded from the analysis [25].

### Spatial microbiome profiling

We used Bowtie2 v2.5.1 to align the spatial transcriptomics read to the GRCh38 human reference genome for human read removal [26]. UMI-tools v1.1.1 was used to separate the fastq files by cell barcode for independent analysis per spots [27]. Bacterial reads were identified using Kraken2 v2.1.1 with the publicly available PlusPF database (version 2022/09/08, https://benlangmead.github.io/aws-indexes/k2) [28]. It contains RefSeq sequences for archaea, bacteria, viruses, plasmids, protozoa, fungi, and humans, as well as UniVec_Core sequences. We calculated counts per million (CPM) mapped reads by dividing bacterial read counts by total sequencing reads for normalization.

### Microbiome profiling in bulk shotgun metagenome data

In the bulk shotgun metagenome data, we employed a process similar to our spatial microbiome detection method. Bowtie2 aligned reads to the GRCh38 human reference genome for human read removal [26]. Subsequently, we identified bacterial reads using Kraken2 with the same PlusPF database used in our spatial profiling analysis [28].

### Spatial microbiome decontamination process

We developed a stepwise decontamination process to reduce false positive species in our spatial microbiome profiling data. Initially, we excluded species with low spatial microbiome sequencing read counts across all samples, specifically those with < 50 total reads across all spatial microbiome sequencing samples. We then compared the profiling results with the bulk metagenome sequencing data from the same tissue samples. Species with < 50 reads from bulk sequencing or with > 1.5 ratio of spatial and bulk sequencing read percentages were removed from our spatial microbiome dataset.

To mitigate potential false positives arising from host read contamination, we evaluated the results of single and double human read removal processes [29]. The single human read removal process used Bowtie2, while the double human read removal process used both Bowtie2 and BWA v0.7.17; all aligned with the GRCh38 human reference genome [26, 30]. This process led to two observations. First, certain species exhibited a drastic reduction in read counts following the double human read removal, indicating that they might be false positives; second, the read counts from genuine gut-residing species also decreased moderately, suggesting that certain true signals were affected (Supplementary Fig. S1). We classified the species as potential false positives if their read counts after double removal dropped to < 2% of those observed after the single removal.

### Comparative analysis of bacterial infiltration

Average number of bacterial reads, in CPM, per spot was compared across the control, CD non-inflamed, and CD inflamed tissues. Number of cells with bacterial infiltration was assessed for each cell type. For each patient, we compared the average number of bacterial reads per spot with their time to relapse and the Rutgeerts score of ileal endoscopic findings [31].

### Differential gene expression and gene set enrichment analysis

Differentially expressed genes were identified based on the presence of specific bacterial species. This analysis was conducted separately for each detected bacteria, and we assessed the consistency of gene expression changes across different bacterial species. Each cell type identified by Cell2location was analyzed, enabling cell type-specific characterization of bacterial-associated transcriptional changes. Gene set enrichment analysis was performed using the BioPlanet 2019 database to identify biological pathways associated with genes consistently expressed differentially in the presence of bacteria [32]. Additionally, we conducted an analysis of tissue-specific gene expression between cells exposed to beneficial and pathogenic microbiomes.

### Quantification of microbial effects on cell viability

We investigated the impact of specific bacterial species on cell viability using the percentage of mitochondrial reads, a widely used marker of cell viability in single-cell RNA sequencing and spatial transcriptomics analyses [33]. Cells with ≤ 10% mitochondrial reads were defined as viable, while those with > 10% were defined as damaged. We calculated the relative risk (RR) of reduced cell viability following exposure to specific bacterial species to quantify the effect of bacterial exposure on cellular integrity. This analysis was performed exclusively on samples from participants with CD. Multiple testing correction was performed using the Benjamini–Hochberg method to control the false discovery rate at 0.05. The RR was calculated as follows:

$$RR=\frac{Number\;of\;damaged\;cells\;exposed\;to\;bacteria/Total\;number\;of\;cells\;exposed\;to\;bacteria}{Number\;of\;damaged\;cells\;not\;exposed\;to\;bacteria/Total\;number\;of\;cells\;not\;exposed\;to\;bacteria}$$

We also calculated Population Attributable Risk Percent (PARP) by integrating the RR values of specific bacteria with their tissue prevalence. This provided a measure of the overall contribution of each bacterial species to cellular damage within the entire tissue environment. The PARP was calculated using the following formula, where *P* is the proportion of cells exposed to the specific bacteria:

$$PARP\;(\%)=\left(\frac{P\times\left(RR-1\right)}{P\times\left(RR-\right)+1}\right)\times100$$

### Correlation analysis of microbiome and host transcriptome

We performed Pearson correlation analysis to evaluate the relationship between the presence of each bacterial species and host gene expression levels. This analysis was performed exclusively on samples from participants with CD. Principal component analysis (PCA) was then applied to the resulting correlation matrix for dimension reduction. We analyzed the contribution of individual genes to principal component 2 (PC2) and displayed the top 20 genes with the highest absolute contribution.

## Results

### Spatial microbiome profiling reveals bacterial localization patterns in pediatric Crohn’s disease

A total of 14 terminal ileal tissue samples were biopsied, including 12 from inflamed and non-inflamed tissues of six children with pediatric ileal CD (mean age 13.5 years, SD 2.1) and two from non-inflamed tissues of two children without CD as the control group (mean age 15.0 years, SD 1.0). Spatial host-microbiome sequencing and bulk shotgun metagenome sequencing were performed on tissues. For the CD group, tissues were collected at the time of diagnosis.

**Fig. 1:**
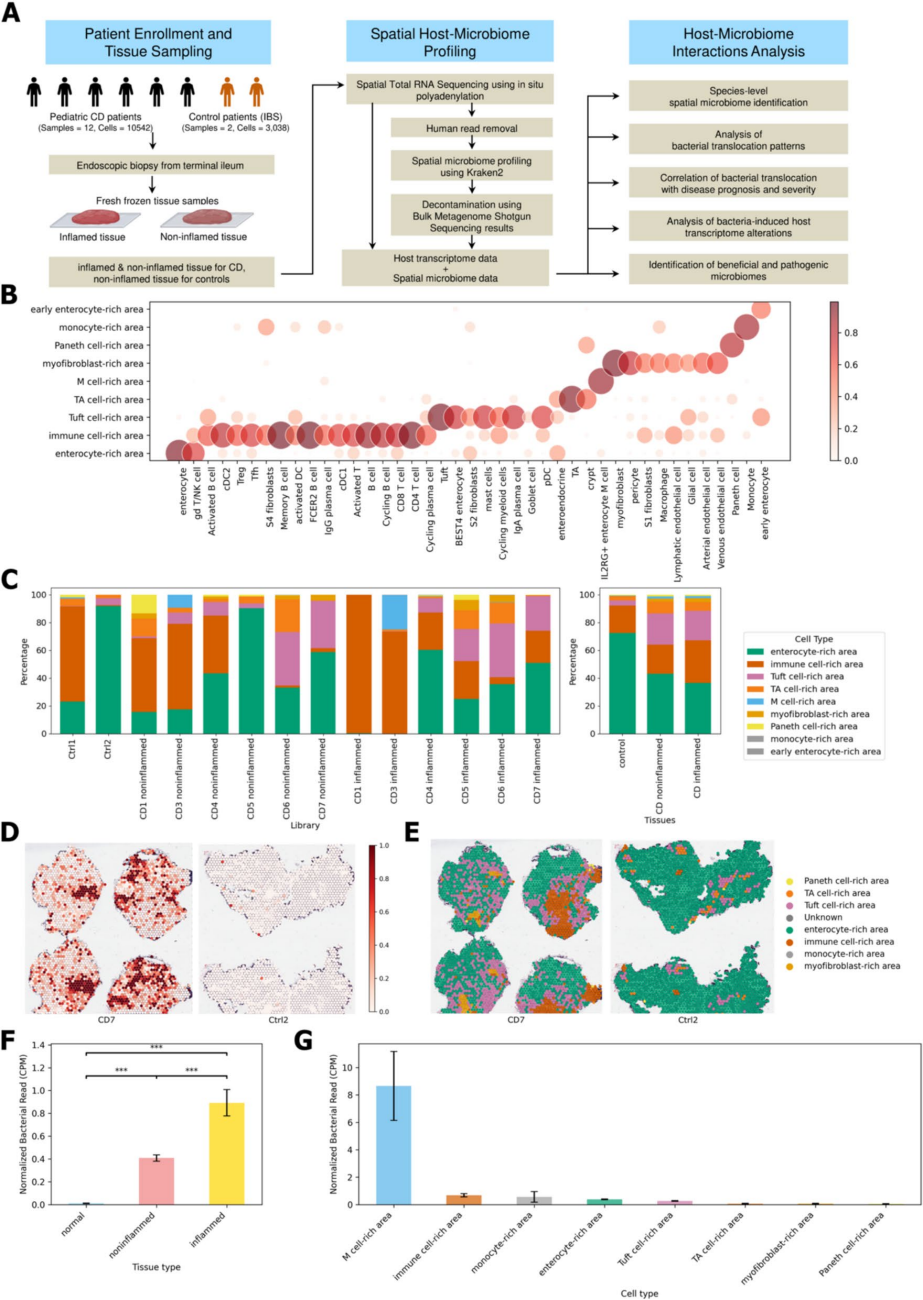
Spatial host-microbiome profiling and bacterial localization patterns in pediatric Crohn’s disease (CD). **A** Schematic illustration of the spatial host-microbiome profiling approach, created using BioRender.com. **B** Cellular composition of areas enriched with various cell types, as identified by Cell2location. **C** Relative abundance of these areas, as identified by Cell2location. **D** Spatial distribution of bacterial read counts (normalized to counts per million mapped reads [CPM]) in tissue sections. The samples shown include CD7 (non-inflamed on the left side of the slide, inflamed on the right side of the slide) and CTRL2. **E** Distribution of cell type-enriched areas in tissue sections corresponding to panel **D**. **F** Average bacterial read count in control, non-inflamed CD, and inflamed CD tissues. Error bars represent standard error of the mean. ∗ *P* < 0.05; ∗ ∗ *P* < 0.005; ∗ ∗ ∗ *P* < 0.0005 (Mann–Whitney two-sided test). **G** Average bacterial read count across different cell type-enriched areas

To capture host RNA and bacterial RNA simultaneously, in-situ polyadenylation was performed using yeast poly(A) polymerase prior to host spatial transcriptome sequencing [17]. Spatial microbiome profiling was conducted by removing human reads using Bowtie2, followed by the identification of bacterial reads using Kraken2 [26, 28]. Bacterial species that were nearly absent in the bulk metagenome shotgun sequencing performed on the same tissue were excluded from the spatial microbiome profiling results. Furthermore, bacterial species that were markedly reduced after additional human read removal using BWA were also excluded, as they were considered potential human read contaminants (Fig. 1A) [30]. As a result, 95 species of bacteria were identified, which are commonly found in the gut.

Cell2location was used to map the spatial distribution of cell types in our spatial transcriptomics data by integrating previously published single-cell RNA sequencing data from pediatric patients with CD [23, 24]. This approach identified various areas enriched with certain cell-types, such as enterocyte-rich, immune cell-rich, and Tuft cell-rich areas (Fig. 1B). Considering the samples from the same participant, the immune cell-rich areas were found to be more abundant in the inflamed CD tissues compared to the non-inflamed tissues (Fig. 1C). The spatial distribution of bacterial reads showed distinct patterns across tissue regions, which were validated by Gram staining of adjacent tissue sections (Fig. 1D, Supplementary Fig. S2 and S3).

Bacterial read count per cell was higher in both non-inflamed (0.409 ± 0.028, mean ± standard error) and inflamed CD tissues (0.892 ± 0.115) compared with that in the controls (0.011 ± 0.002), with the highest count observed in inflamed CD tissues (Fig. 1E, G). Notably, we observed increased counts in M cell-rich areas (8.660 ± 2.516) (Fig. 1H), consistent with the known function of M cells in sampling luminal bacteria [34].

### Bacterial abundance predicts Crohn’s disease prognosis

**Fig. 2:**
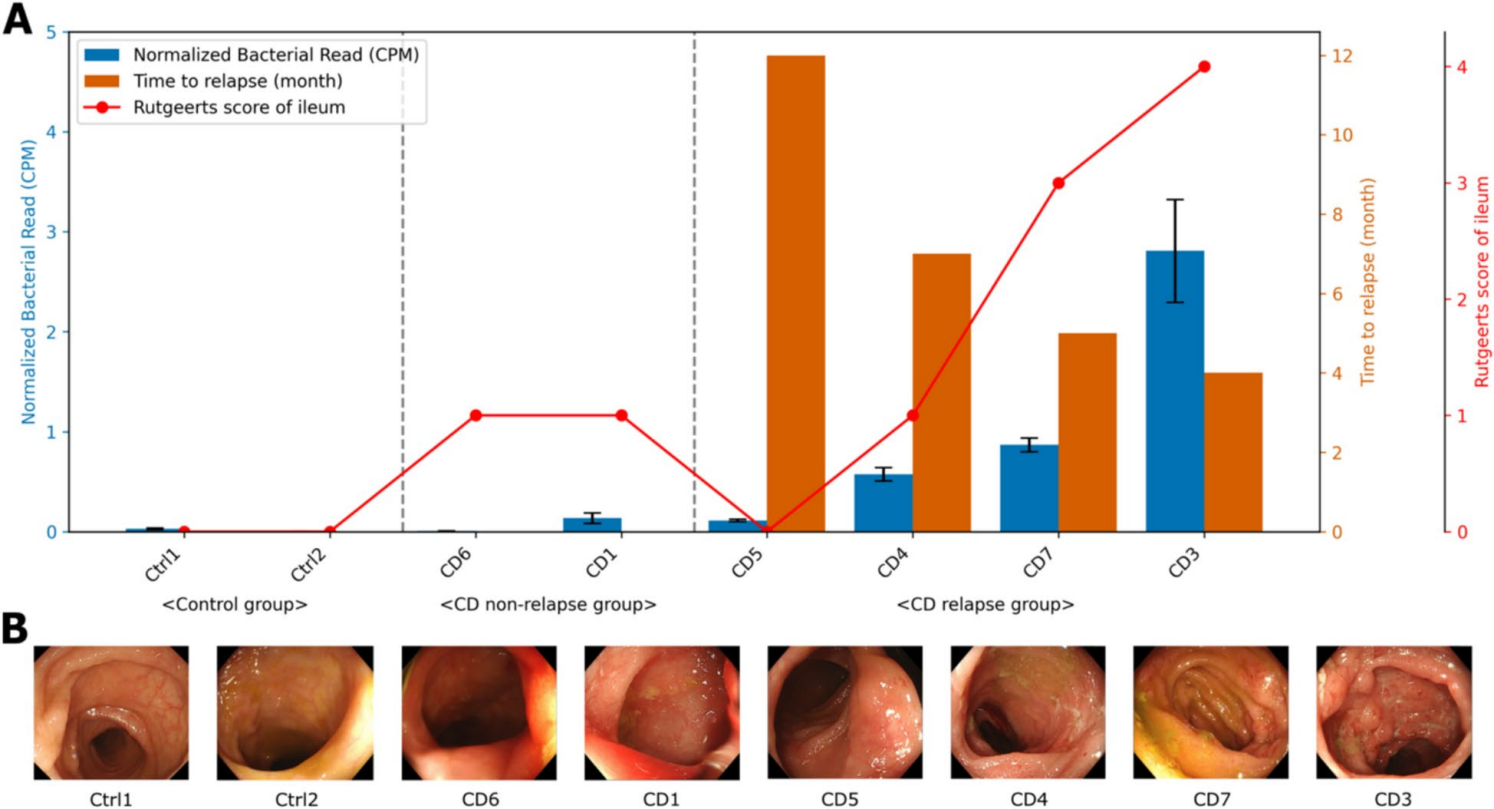
Correlation of bacterial abundance with prognostic indicators and ileal endoscopic findings in pediatric Crohn’s disease (CD). **A** Patients were categorized into three groups: Control (Ctrl), CD without relapse, and CD with relapse. Within each group, individuals were arranged by increasing bacterial read count. The blue bars (left *y*-axis) show the average bacterial read count, normalized to counts per million mapped reads (CPM) for each patient. The orange bars (right *y*-axis) represent the time to relapse (in months) for patients with CD who experienced relapse. The red line indicates the Rutgeerts score, which assesses the severity of endoscopic findings in CD (ranging from i0 [no lesions] to i4 [diffuse inflammation with large ulcers]). Error bars represent the standard error of the mean. **B** Endoscopic images of the terminal ileum in patients with CD are presented, corresponding to the order of patients in panel **A**

The bacterial read count per cell was highest in the CD group with relapse, followed by the CD group without relapse, and lowest in the control group. Within the relapse group, the time to relapse was shorter for participants who had a higher count at the time of diagnosis (Fig. 2A). We also observed an association between bacterial read counts and the severity of endoscopic findings in the ileum, which was assessed by the Rutgeerts score (Fig. 2B) [31]. These findings suggest the potential for predicting CD prognosis by assessing the extent of bacterial translocation in intestinal tissues.

### Bacterial presence activates host immune responses

**Fig. 3:**
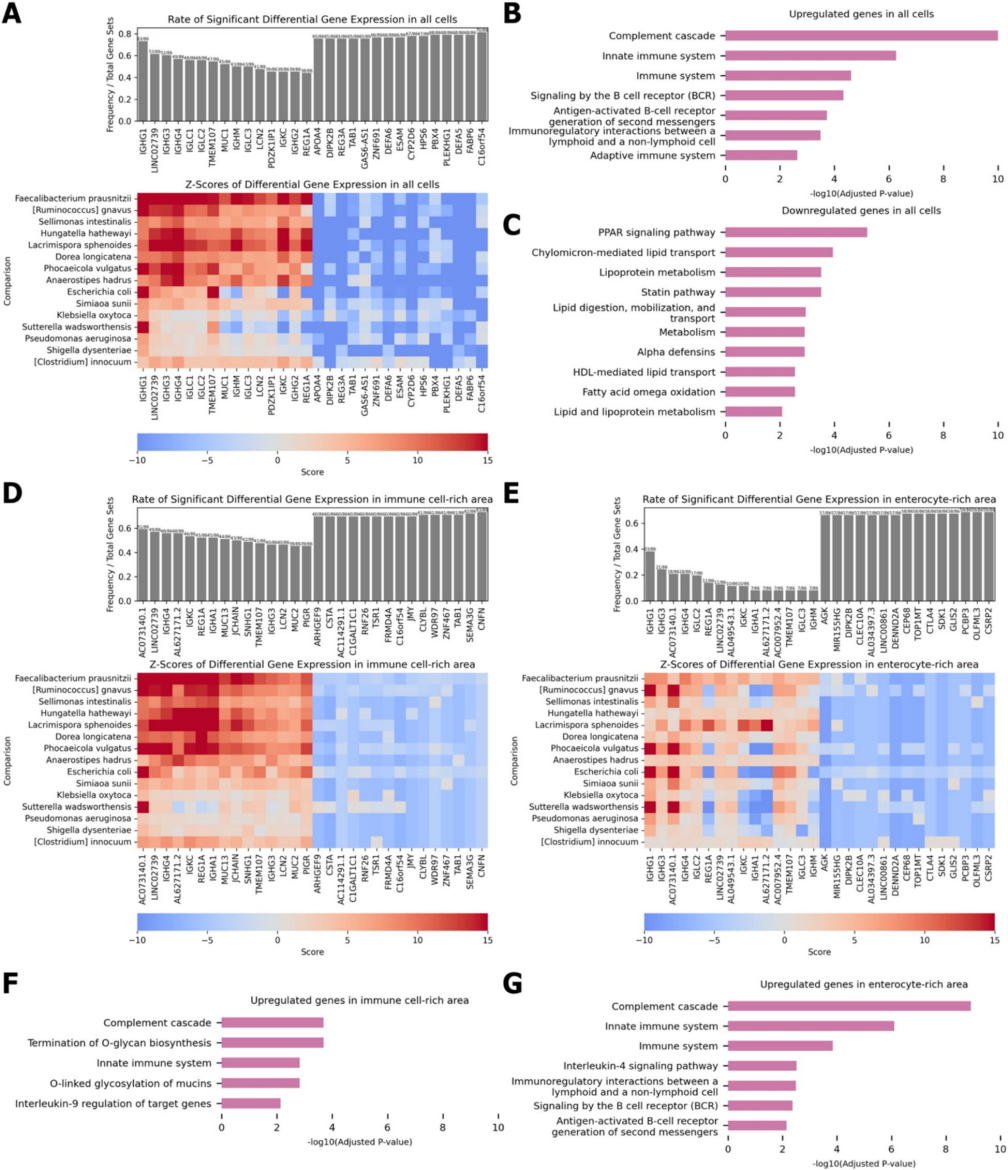
Differential gene expression (DGE) analysis in response to bacterial presence in Crohn’s disease. **A** DGE analysis in all cells. The top panel shows the rate of significant DGE in response to various bacteria. The heatmap below displays *Z*-scores of DGE for various bacterial species. **B** Gene set enrichment analysis (GSEA) of upregulated genes in all cells in response to bacterial presence. **C** GSEA of downregulated genes in all cells in response to bacterial presence. **D** DGE analysis in immune cell-rich areas. **E** DGE analysis in enterocyte-rich areas. **F** GSEA of upregulated genes in immune cell-rich areas in response to bacterial presence. **G** GSEA of upregulated genes in enterocyte-rich areas in response to bacterial presence

Genes differentially expressed based on the presence of 95 bacterial species were identified. Consistency of this expression pattern was examined across different bacterial species to identify genes whose expression was consistently induced or suppressed by the presence of bacteria. Furthermore, using the BioPlanet database, we investigated the functions of gene that showed consistent overexpression [32]. Gene expression analysis in all cells showed that bacterial presence induced upregulation of immune system components, including B cell receptor signaling (Fig. 3A, B). Meanwhile, lipid absorption pathway of the intestine, such as chylomicron-mediated lipid transport, were downregulated in the presence of bacteria (Fig. 3C).

Gene expression analysis of immune cell-rich and enterocyte-rich areas demonstrated consistent upregulation of genes encoding immunoglobulin components in response to bacterial presence, including *IGHG4*, *IGKC*, *IGHA1*, *JCHAIN*, and *IGHG3* in immune cell-rich areas and *IGHG1*, *IGHG3*, *IGHG4*, *IGLC2*, *IGKC, IGHA1*, *IGLC3*, and *IGHM* in enterocyte-rich areas (Fig. 3D, E) [35]. Functional enrichment analysis revealed that upregulated genes in both areas were involved in bacterial defense mechanisms, including interleukin signaling and innate immune system pathway (Fig. 3F, G) [32]. Moreover, the genes in the immune cell-rich areas showed a higher degree of consistency in their differential expression compared with those in the enterocyte-rich areas, suggesting that the transcriptional response of immune cells to bacterial presence is more uniform and robust, underlining their central role in the bacterial defense response.

### Bacterial species distinctly impact cell viability in Crohn’s disease

**Fig. 4:**
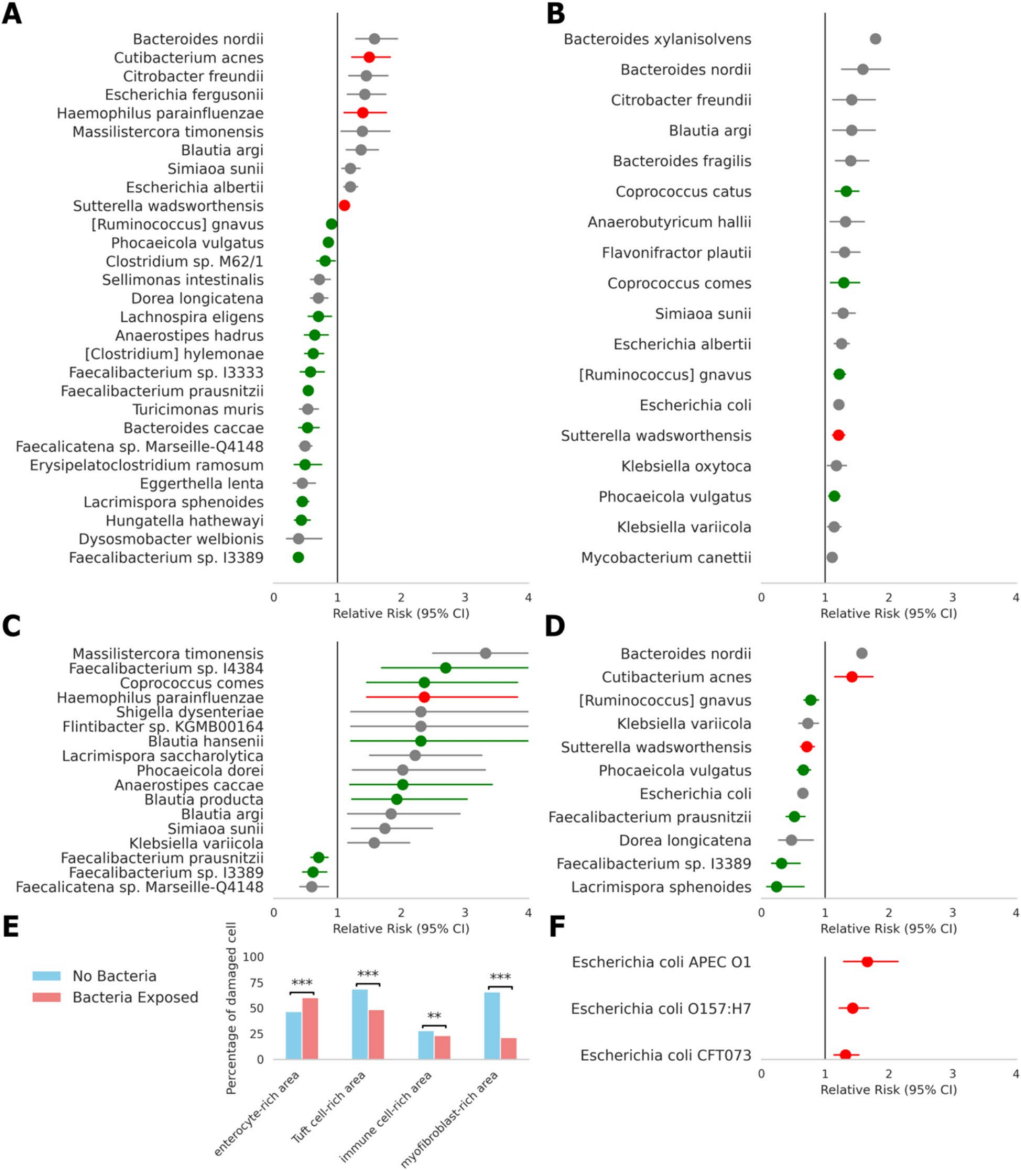
Relative risks (RR) of reduced cell viability associated with exposure of various bacterial species in pediatric Crohn’s disease (CD). **A**–**D** RR of reduced cell viability upon exposure to various bacteria in **A** all cells, **B** enterocyte-rich areas, **C** immune cell-rich areas, and **D** tuft cell-rich areas. Statistically significant results are presented (Benjamini–Hochberg method, significance threshold of 0.05). Green dots indicate bacteria previously reported to decrease CD risk, while red dots indicate bacteria known to increase CD risk. Error bars represent 95% confidence intervals. **E** Comparison of the fraction of damaged cells (> 10% mitochondrial reads) between bacteria-exposed and non-exposed cells across different cell types. ∗ *P* < 0.05; ∗ ∗ *P* < 0.005; ∗ ∗ ∗ *P* < 0.0005 (Mann–Whitney two-sided test). **F** RR of reduced cell viability for various *Escherichia coli* strains

The RR values of reduced cell viability were calculated following exposure to specific bacterial species. We performed multiple testing corrections using the Benjamini–Hochberg method and visualized significant results after this correction. This analysis was performed solely on samples from participants with CD. The RR values and their 95% confidence intervals were displayed (Fig. 4A, Supplementary Table S2). Based on Supplementary Table S3, species previously reported to increase CD risk are shown in red, while those known to decrease it are shown in green [9, 10]. This demonstrates that previously known beneficial and pathogenic microbiomes were consistently observed in our study.

*Bacteroides caccae* (RR = 0.53; 95% confidence interval [CI], 0.39–0.71), *F. prausnitzii* (RR = 0.54; 95% CI, 0.49–0.60), *Phocaeicola vulgatus* (RR = 0.85; 95% CI, 0.80–0.92), and *Ruminococcus gnavus* (RR = 0.91; 95% CI, 0.84–0.98) were confirmed as beneficial microbes, aligning with previous findings. *Anaerostipes hadrus* (RR = 0.64; 95% CI, 0.48–0.85), *Hungatella hathewayi* (RR = 0.43; 95% CI, 0.33–0.57), *Faecalibacterium sp*. *I3333* (RR = 0.57; 95% CI, 0.41–0.79), and *sp. I3389* (RR = 0.38; 95% CI, 0.31–0.47) were previously identified as beneficial at the genus level, even though their species-level association with CD were not reported. *Cutibacterium acnes* (RR = 1.50; 95% CI, 1.22–1.83), *H. parainfluenzae* (RR = 1.40; 95% CI, 1.10–1.17), and *Sutterella wadsorthensis* (RR = 1.11; 95% CI, 1.03–1.19) were confirmed as pathogenic microbes at the species level, consistent with prior studies.

We also calculated the PARP by integrating the RR values of specific bacteria with their gut prevalence. This measured the overall contribution of each bacterial species to intestinal barrier disruption within the entire gut environment. *F. prausnitzii*, a highly abundant gut bacterium known for its beneficial properties in CD, was the most influential in reducing intestinal barrier disruption (by − 4.6%) (Supplementary Fig. S4).

When the RR was examined specifically in enterocyte-rich areas (Fig. 4B), all bacteria were found to reduce cell viability, even species such as *Phocaeicola vulgatus*, which was initially found to inhibit this reduction during analysis of the entire cell population. In contrast, analyses of immune cell- and tuft cell-rich areas revealed that some bacteria exhibited a protective effect by inhibiting the reduction in cell viability (Fig. 4C, D). Additionally, bacterial exposure increased the fraction of damaged cells in enterocyte-rich areas, but decreased it in tuft cell-rich, immune cell-rich, and myofibroblast-rich areas. This suggests that the beneficial effect of certain gut microbiomes may be facilitated through interactions with cell types other than enterocytes, potentially including immune and tuft cells. These findings highlight the complex interplay between various host gut cell types and the resident bacteria.

To further investigate cell type-specific bacterial associations, we calculated co-existence frequencies between each bacterial species and 42 cell types by multiplying Cell2location-derived cell type fractions with bacterial counts per spot (Supplementary Fig. S5). This analysis revealed that beneficial microbiome candidates were particularly enriched in M cells and memory B cells, while pathogenic microbiome candidates showed higher prevalence in enterocytes and tuft cells.

To account for the heterogeneity of *Escherichia coli*, a highly abundant species with diverse strains ranging from commensal to pathogenic, we calculated the RR values for 42 different *E. coli* strains characterized at the strain level, although only 15.2% were identified at this taxonomic resolution (Fig. 4F, Supplementary Fig. S6, Supplementary Table S4). We identified *E. coli APEC O1* (RR = 1.66; 95% CI, 1.29–2.14), *O157:H7* (RR = 1.43; 95% CI, 1.22–1.68), and *CFT073* (RR = 1.32; 95% CI, 1.14–1.53) as part of the pathogenic microbiome, which is consistent with previous reports [36–38].

### Beneficial and pathogenic microbiomes distinctly modulate host transcription

We analyzed differentially expressed genes between cells exposed to beneficial and pathogenic microbiomes. Using 16 beneficial and nine pathogenic members identified in this study, we formed 144 beneficial-pathogenic microbiome pairs. For each pair, we compared gene expressions in response to the beneficial versus pathogenic microbiome. We assessed the consistency of these changes across the different pairs, identifying genes consistently upregulated or downregulated in the presence of beneficial or pathogenic bacteria.

**Fig. 5:**
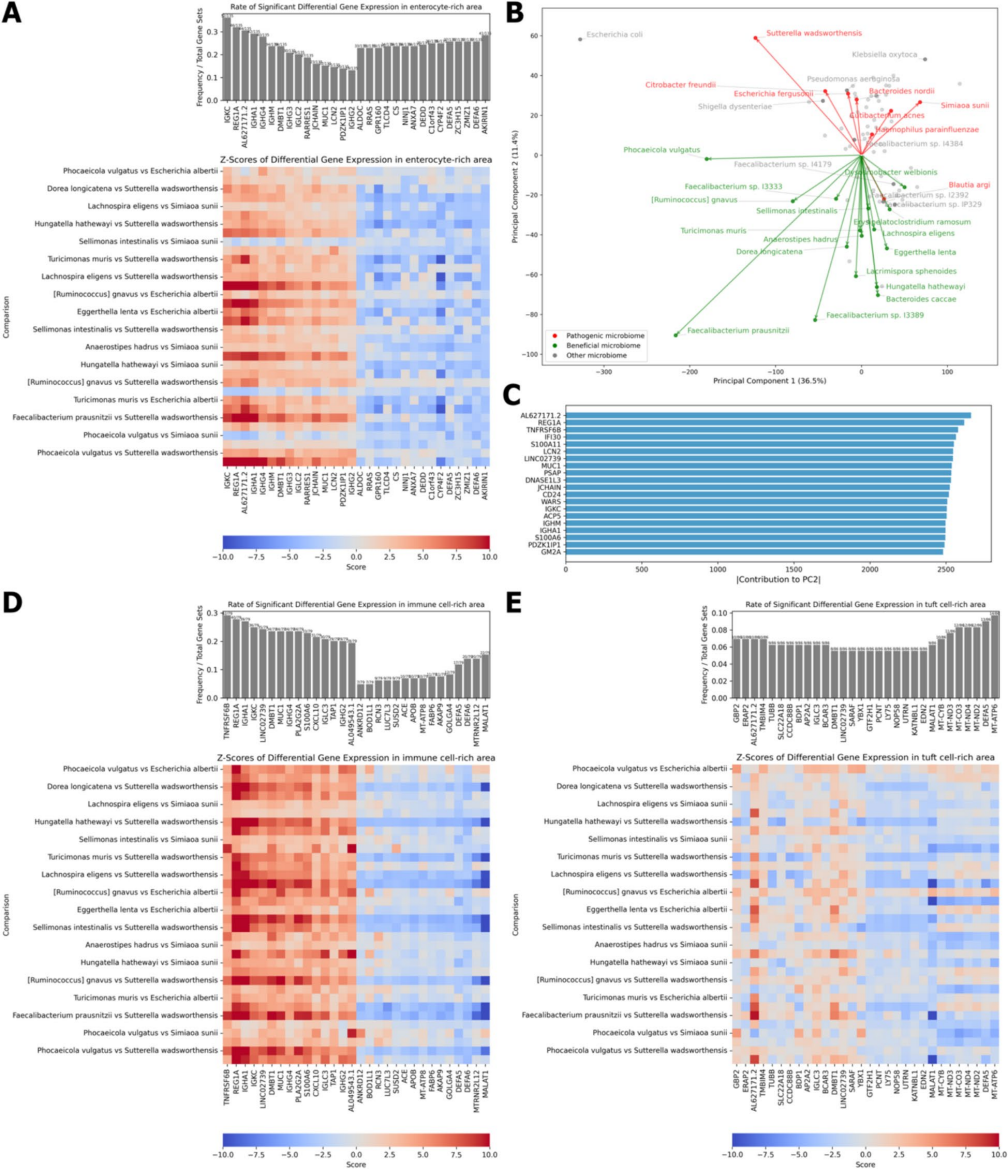
Differential gene expression (DGE) analysis between cells exposed to beneficial and pathogenic microbiomes and Principal Component Analysis (PCA) of the correlation matrix between bacterial presence and host gene expression in pediatric Crohn’s disease. **A**, **D**, **E** DGE analysis in **A** all cells, **D** immune cell-rich areas, and **E** enterocyte-rich areas. The top panel shows the rate of significant DGE between cells exposed to beneficial and pathogenic microbiome members. The heatmaps display *Z*-scores of DGE. **B** PCA of the correlation matrix between bacterial presence and host gene expression levels. Pathogenic microbiome members are shown in red, and beneficial microbiome members are shown in green. **C** Bar chart displaying the top 20 genes with the highest absolute contribution to Principal Component 2

Across all cells, beneficial microbiomes increased the expression of immunoglobulin genes, including *IGHG3*, *IGHG4*, *IGLC1*, *IGHM*, *IGKC*, *IGLC2*, *IGHG2*, and *IGHA1* (Fig. 5A) [35]. This suggests that beneficial microbiomes may exert their effects through immune cells. In immune cell-rich areas, genes such as *REG1A* and *TNFRSF6B* showed increased expression in regions with beneficial microbiomes (Fig. 5D). Similarly, in enterocytes, genes including *IGKC* and *REG1A* were upregulated in such areas (Fig. 5E).

To assess species-specific bacterial effects on host gene expression, we calculated Pearson correlations for bacterial presence and gene expression levels. We then applied PCA for dimension reduction of the correlation matrix. The results showed a clear distinction between beneficial and pathogenic microbial species along PC2 (Fig. 5B). Furthermore, the expression of genes such as *REG1A* and *TNFRSF6B* had substantial contributions in PC2 (Fig. 5C, Supplementary Fig. S7).

## Discussion

The critical roles of bacteria-host interactions in CD are increasingly acknowledged. However, the intricate relationships between various cell types and bacterial species at the cellular level are still poorly understood [1, 4, 11]. Therefore, we developed a novel spatial host-microbiome profiling approach that, to the best of our knowledge, is the first to enable simultaneous species-level identification of bacteria and host transcriptomics. Using this method, we demonstrated increased bacterial presence in pediatric CD tissues, with a significant association between bacterial abundance and disease prognosis, while also revealing distinct host transcriptome alterations in response to various bacterial species. Furthermore, we identified and characterized potentially beneficial and pathogenic microbial species associated with CD, including several newly discovered risk-modulating bacterial species. Our spatial host-microbiome profiling approach not only provides profound insights regarding CD, but also offers potential applications for studying various microbiome-associated diseases such as gastrointestinal cancer and infectious diseases. It reveals spatial associations between gut bacteria and various host cell types in CD pathophysiology at the cellular level. Moreover, the identification of beneficial and pathogenic microbiome members enables the development of microbiome-based therapeutic strategies.

Our results highlighted the importance of bacterial presence in CD, revealing that CD tissues exhibited increased bacterial abundance compared with controls. Furthermore, within CD samples, inflamed tissues showed higher bacterial abundance compared with non-inflamed tissues. These findings suggest a more active bacterial invasion in CD, potentially due to impaired host defense mechanisms [39], which aligns with the findings of Sun, D, et al., who also reported an increase in bacterial translocation in inflamed tissues compared with non-inflamed tissues in CD [39]. Moreover, the bacterial abundance at diagnosis not only predicted disease prognosis in patients with CD but also showed a strong association with the severity of endoscopic findings in the ileum. This indicates that the level of bacterial infiltration in intestinal tissues could serve as a potential prognostic marker for predicting disease course in pediatric CD.

Analysis of differentially expressed genes in response to the presence of bacterial species revealed a specific and targeted response to bacterial infiltration. Consistent upregulation of genes encoding immunoglobulin components appears to be a response to bacterial presence, which was further confirmed by gene enrichment analysis revealing the involvement of these upregulated genes in bacterial defense functions. Notably, the higher degree of consistency in the differential expression of genes in immune cell-rich areas compared with enterocyte-rich areas shows the uniform and robust transcriptional response of immune cells to bacterial presence. Furthermore, our data showed the upregulation of lncRNA, such as LINC02739, in response to bacterial infiltration, suggesting their potential involvement in the host-microbe interaction and disease pathogenesis. While the specific functions of these lncRNAs remain unclear, their differential expression warrants further investigation into their roles in modulating the immune response and disease progression in CD [40].

To identify potentially beneficial and pathogenic microbes in pediatric CD, we assessed the impact of specific bacterial species on intestinal barrier integrity by determining the RRs of reduced cell viability upon exposure to these bacteria. Our analysis identified 16 beneficial and nine pathogenic microbiome candidates. Among these, six were previously reported to have a confirmed impact on CD at the species level, while eight were known to affect only at the genus level [9, 10]. Interestingly, the majority of the beneficial microbiome members we identified (13 out of 16 species) belong to *Bacillota* (*Firmicutes*), with a substantial number (11 species) belonging to *Clostridium* clusters XIVa and IV, which have been previously associated with a reduced risk of CD (Supplementary Table S2) [41–50]. In contrast, five out of nine pathogenic microbiome members, including *Citrobacter freundii*, *Escherichia fergusonii*, *H. parainfluenzae*, *Escherichia albertii*, and *Sutterella wadsworthensis*, belong to *Pseudomonadota* (*Proteobaccteria*). This aligns with previous studies suggesting an association between increased abundance of *Pseudomonadota* (*Proteobaccteria*) and CD [11, 51]. Notably, we identified five beneficial microbiome members that were not reported in previous studies. Four of these newly identified beneficial microbes belong to *Bacillota* (*Firmicutes*), showing similar phylogeny to other known beneficial microbes. Interestingly, three of these newly identified species, *Sellimonas intestinalis*, *Turicimonas muris*, and *Dysosmobacter welbionis*, have been reclassified or newly discovered after the publication of the reference studies we used (Gevers et al., 2014; Kansal et al., 2019) [9, 10, 43, 50, 52]. These findings demonstrate that our newly identified beneficial microbes are aligned with those from previous reports and highlight how our understanding of the microbiome deepens as bacterial databases expand and taxonomic classifications become more precise.

Our study highlights that the impact of gut microbes on CD pathogenesis is not uniform, even within the same genus, as demonstrated by the *Faecalibacterium* genus. While *F. prausnitzii*, *Faecalibacterium sp. I3389*, and *Faecalibacterium sp. I3333* were identified as beneficial microbes in this study, *Faecalibacterium sp. I2392*, *Faecalibacterium sp. I4179*, *Faecalibacterium sp. I4384*, and *Faecalibacterium sp. IP329* did not have statistically significant RR values. Additionally, our spatial microbiome analysis at the strain level confirmed that *E. coli*, which has various strains ranging from commensal to pathogenic, showed varying associations with CD risk [36–38, 53].

Comparing tissue-specific gene expression between cells exposed to beneficial and pathogenic microbiomes, we observed increased expression of genes such as *REG1A* and *TNFRSF6B* in the presence of beneficial microbiomes. According to Mao et al., *REG1A* plays a crucial role in tissue regeneration and the repair of intestinal epithelial damage [54]. In their study, inducing *REG1A* expression in a DSS colitis mouse model promoted the recovery of the intestinal barrier. TNFRSF6B, also known as DcR3, is a well-known inhibitor of FASL and LIGHT, which are essential for cell apoptosis [55]. The upregulation of *TNFRSF6B* in the presence of beneficial bacteria suggests that these microbes may be associated with the maintenance of intestinal epithelial integrity, potentially through their influence on host cell apoptosis pathways. These findings provide insights into the potential mechanisms by which beneficial microbiomes may provide protection against CD pathogenesis, emphasizing the need for further research to understand the complex interactions between gut microbes and host cellular processes.

Our spatial host-microbiome sequencing approach offers several advantages over traditional methods. Unlike dissociation-based single-cell RNA sequencing methods such as Chromium, our approach allows for the accurate identification of host cells exposed to bacteria, as it avoids the potential dissociation of bacteria and host cells during sample preparation [56]. Additionally, our method minimizes the risk of contamination by ambient RNA, a common issue in droplet-based methods, resulting from cell lysis and RNA release within the microfluidic droplets, ensuring that the detected bacterial reads accurately reflect the true bacterial distribution within the tissue [57]. Furthermore, our approach enables the analysis of damaged cells, which are typically removed in dead cell removal processes prior to single-cell RNA sequencing studies [33]. This is considerable in the context of CD, where removing damaged cells may inadvertently exclude the more inflamed portion of the tissue, leading to a potential bias in the results.

Previous studies using 16 s rRNA targeting approaches identified bacterial distributions only at the genus level [58]. In contrast, the Kraken2-based shotgun metagenomic profiling approach employed in our study allows for the detection of even small amounts of bacteria and enables differentiation at the strain level [28]. Moreover, our approach eliminates the need for creating probes, allowing for the evaluation of a wide range of bacteria, including those less frequently reported in the literature, some of which may have potential as therapeutic targets [18–20]. Unlike probe-based methods that restrict analysis to pre-selected bacterial targets, our shotgun metagenomic approach enables comprehensive identification of all bacterial species present in the sample. This is achieved through a simple sample preparation step with the addition of yeast poly(A) polymerase, eliminating the need for the cumbersome preparation of new probes for specific bacteria.

This study has several limitations. First, the small sample size and limited age range may introduce biases, although the total number of cells analyzed was substantial (13,876). Future studies should include more samples from diverse ethnic backgrounds to improve generalizability. Second, our control group consisted of pediatric IBS patients rather than healthy individuals, as obtaining intestinal biopsies from healthy children presents ethical and practical challenges. We selected patients who volunteered for endoscopy and showed no inflammatory markers on blood tests, endoscopic examination, or histopathological analysis. Third, the spatial transcriptomic technology lacks single-cell resolution, resulting in the mixing of various cell types within spots [23]. Although we employed Cell2location to classify areas based on cell combinations, heterogeneity within areas of the same cell-type may still exist, meaning that there could be slight differences in cellular composition among areas classified as the same type. This heterogeneity could potentially influence the comparisons made within these areas, such as those for differentially expressed genes in immune cell-rich areas due to bacterial exposure. However, the emergence of high-resolution single-cell spatial transcriptomics techniques offers opportunities to apply our algorithm to more refined spatial data in future investigations, which could help reduce the impact of cellular heterogeneity on our analyses [59].

Additionally, future studies should incorporate diverse modalities including metabolomics data and leverage various analytical methods and multi-omics databases to comprehensively characterize the complex interactions between bacteria and host responses in CD [60]. Improvements in reference databases, more sophisticated taxonomic classification algorithms, and enhanced spatial transcriptomics methods for bacterial RNA capture will be essential for further advancement and broader applications of spatial microbiome profiling. Moreover, experimental validation of the beneficial microbiomes and host response mechanisms identified in this study will be important research directions.

## Conclusions

In conclusion, our study introduces a novel spatial host-microbiome profiling approach that enables the simultaneous profiling of the host transcriptome and bacterial species at a high taxonomic resolution in the ileal tissues of pediatric patients with CD. This approach allowed us to identify increased bacterial presence in CD tissues, as well as the potential prognostic value of assessing bacterial infiltration in intestinal tissues. We also discovered specific beneficial and pathogenic microbiomes associated with CD pathogenesis and suggested potential mechanisms by which these microbes may influence disease progression, such as the modulation of host cell apoptosis pathways. The identification of several newly discovered beneficial microbiomes provides promising candidates for the development of novel microbiome-based therapeutics for CD. Our spatial host-microbiome sequencing approach offers a valuable method to understand bacterial-associated host transcriptional alterations in the context of CD pathogenesis.

## Supplementary Information

Additional file 1: STROBE Statement—checklist of items that should be included in reports of observational studies.

Additional file 2: Supplementary methods. Supplementary Figure S1 Comparison of spatial versus bulk sequencing detection and single versus double human read removal for gut-residing and false positive bacterial species (A, B) Comparison between spatial sequencing and bulk shotgun sequencing detection for genuine gut-residing species (Faecalibacterium prausnitzii and Escherichia coli) and potential false positive species (Guillardia theta and Puccinia striiformis). (C, D) Comparison of bacterial read counts between single (Bowtie2 only) and double (Bowtie2 + BWA) human read removal processes for genuine gut-residing species (Faecalibacterium prausnitzii and Escherichia coli) and potential false positive species (Guillardia theta and Puccinia striiformis). Supplementary Figure S2 Validation of spatial microbiome profiling by Gram staining of adjacent tissue sections. (A) Spatial distribution of bacterial reads (red intensity) across tissue sections from CD patients and controls, as detected by spatial host-microbiome profiling. (B) Histological appearance (H&E staining) of corresponding tissue sections showing tissue architecture. (C) Gram staining of adjacent tissue sections demonstrating bacterial localization patterns with Gram-positive bacteria appearing as dark purple clusters. Supplementary Figure S3 High-magnification correlation between spatial microbiome sequencing and Gram staining. (A-C) Higher magnification views of selected regions showing correlation between spatial sequencing bacterial signals and Gram-positive bacterial clusters (dark purple) within tissue structures. The spatial distribution patterns of bacteria detected by sequencing show overall similarity with bacterial localization observed in Gram-stained adjacent sections, validating the spatial microbiome profiling approach. Supplementary Figure S4 Population Attributable Risk Percent (PARP) analysis of bacterial species in pediatric Crohn's disease. PARP values for bacterial species, calculated by integrating relative risk values with tissue prevalence. Green bars indicate beneficial bacteria that reduce intestinal barrier disruption, while red bars represent pathogenic bacteria that increase tissue damage. Supplementary Figure S5 Cell type-specific co-localization probabilities of bacterial species in pediatric ileal Crohn's disease tissues. (A) Heatmap showing co-localization probabilities between bacterial species and immune cell types. Color intensity represents the strength of co-localization probability calculated by multiplying Cell2location-derived cell type fractions with bacterial counts per spot. (B) Co-localization probabilities between bacterial species and non-immune cell types. (C) Log2 fold change comparison of co-localization probabilities between beneficial and pathogenic microbiomes across different cell types. Green bars indicate cell types with higher co-localization probability for beneficial microbiomes, while red bars indicate higher probability for pathogenic microbiomes. Statistical significance is indicated by asterisks (*P < 0.05, **P < 0.01, ***P < 0.001). Supplementary Figure S6 Strain-level relative risk analysis of bacterial species associated with reduced cell viability in pediatric Crohn's disease. Relative risk (RR) values for strain-level bacterial species and their association with reduced cell viability in CD tissues. Red circles indicate pathogenic strains with RR > 1.0, while gray circles reƒpresent beneficial strains with RR < 1.0. Error bars represent 95% confidence intervals. Only statistically significant results after Benjamini–Hochberg correction are shown. Supplementary Figure S7 Gene contribution to Principal Component 1 in bacterial presence correlation analysis. Bar chart displaying the top 20 genes with the highest absolute contribution to Principal Component 1 (PC1) in the correlation analysis between bacterial presence and host gene expression levels.

Additional file 3: Supplementary Table S1. Baseline clinical, laboratory, and endoscopic characteristics of pediatric CD patients and IBS control subjects. Supplementary Table S2. Bacterial relative risk analysis for reduced cell viability in pediatric Crohn's disease. Supplementary Table S3. Previous literature reports of bacterial associations with Crohn's disease risk according to Gevers et al. (2014) and Kansal et al. (2019). Supplementary Table S4. Strain-level bacterial relative risk values for reduced cell viability in pediatric Crohn's disease.

## Data Availability

The raw sequencing data generated in this study have been deposited in the NCBI Sequence Read Archive (SRA) under the accession number PRJNA1159478. Prior to publication, the data can be accessed at https://dataview.ncbi.nlm.nih.gov/object/PRJNA1159478?reviewer=sjgo3s8uv1itu8c37csovv3qc5.

## Abbreviations

CD: Crohn’s disease)
RR: Relative risk
RIN: RNA Integrity Number
PARP: Population Attributable Risk Percent
