# Supplementary material for: Spatial host-microbiome profiling demonstrates bacterial-associated host transcriptional alterations in pediatric ileal Crohn’s disease
Source: Microbiome. 2025 Aug 23;13:189. doi: 10.1186/s40168-025-02178-8 (PMC12374449; doi:10.1186/s40168-025-02178-8)
Supplement: Supplementary file 2 — Additional file 1: STROBE Statement—checklist of items that should be included in reports of observational studies. [file 40168_2025_2178_MOESM1_ESM.docx]

STROBE Statement—checklist of items that should be included in reports of observational studies

|  | Item No. | Recommendation | Page  No. | Relevant text from manuscript |
| --- | --- | --- | --- | --- |
| **Title and abstract** | 1 | (*a*) Indicate the study’s design with a commonly used term in the title or the abstract | 3 | We analyzed 14 terminal ileal tissue samples from six pediatric CD patients and two controls in this prospective case-control study. |
|  |  | (*b*) Provide in the abstract an informative and balanced summary of what was done and what was found | 3 |  |
| Introduction | | | |  |
| Background/rationale | 2 | Explain the scientific background and rationale for the investigation being reported | 6, 7 |  |
| Objectives | 3 | State specific objectives, including any prespecified hypotheses | 7 | To overcome these limitations and advance our understanding of CD pathogenesis, we present a spatial host-microbiome profiling approach that allows for species-level identification of bacteria while simultaneously capturing host transcriptomics. Using this method, we examine bacterial translocation patterns in CD tissues and their association with host transcriptome alterations at cellular resolution. Additionally, we identify and characterize microbiomes potentially associated with beneficial of pathogenic effects in CD. This approach provides insights into CD pathophysiology at the cellular level and offers potential applications for understanding host-microbiome interactions in various microbiome-associated diseases. |
| Methods | | | |  |
| Study design | 4 | Present key elements of study design early in the paper | 8 | Participant and ethics |
| Setting | 5 | Describe the setting, locations, and relevant dates, including periods of recruitment, exposure, follow-up, and data collection | 8 | Participant and ethics |
| Participants | 6 | (*a*) *Cohort study*—Give the eligibility criteria, and the sources and methods of selection of participants. Describe methods of follow-up  *Case-control study*—Give the eligibility criteria, and the sources and methods of case ascertainment and control selection. Give the rationale for the choice of cases and controls  *Cross-sectional study*—Give the eligibility criteria, and the sources and methods of selection of participants | 8 | Participant and ethics |
|  |  | (*b*) *Cohort study*—For matched studies, give matching criteria and number of exposed and unexposed  *Case-control study*—For matched studies, give matching criteria and the number of controls per case | 8 | Participant and ethics |
| Variables | 7 | Clearly define all outcomes, exposures, predictors, potential confounders, and effect modifiers. Give diagnostic criteria, if applicable | 8, 9, 10 | Sample collection and storage |
| Data sources/ measurement | 8* | For each variable of interest, give sources of data and details of methods of assessment (measurement). Describe comparability of assessment methods if there is more than one group | 8 | Sample collection and storage |
| Bias | 9 | Describe any efforts to address potential sources of bias | 11 | Spatial microbiome decontamination process |
| Study size | 10 | Explain how the study size was arrived at | 8 | Sample collection and storage |

Continued on next page

| Quantitative variables | 11 | Explain how quantitative variables were handled in the analyses. If applicable, describe which groupings were chosen and why | 10, 11, 12, 13 | Host transcriptome analysis,  Spatial microbiome profiling,  Microbiome profiling in bulk shotgun metagenome data,  Comparative analysis of bacterial infiltration,  Differential gene expression and gene set enrichment analysis,  Quantification of microbial effects on cell viability,  Correlation analysis of microbiome and host transcriptome |
| --- | --- | --- | --- | --- |
| Statistical methods | 12 | (*a*) Describe all statistical methods, including those used to control for confounding | 12, 13 | Quantification of microbial effects on cell viability,  Correlation analysis of microbiome and host transcriptome |
|  |  | (*b*) Describe any methods used to examine subgroups and interactions | 12, 13 | Quantification of microbial effects on cell viability,  Correlation analysis of microbiome and host transcriptome |
|  |  | (*c*) Explain how missing data were addressed | - | There was no missing data in this study. |
|  |  | (*d*) *Cohort study*—If applicable, explain how loss to follow-up was addressed  *Case-control study*—If applicable, explain how matching of cases and controls was addressed  *Cross-sectional study*—If applicable, describe analytical methods taking account of sampling strategy | 8, 12, 13 | Sample collection and storage, Quantification of microbial effects on cell viability,  Correlation analysis of microbiome and host transcriptome |
|  |  | (*e*) Describe any sensitivity analyses | 12, 13 | Quantification of microbial effects on cell viability,  Correlation analysis of microbiome and host transcriptome |
| Results | | | | |
| Participants | 13* | (a) Report numbers of individuals at each stage of study—eg numbers potentially eligible, examined for eligibility, confirmed eligible, included in the study, completing follow-up, and analysed | 14 | Figure1a |
|  |  | (b) Give reasons for non-participation at each stage | 8 | Sample collection and storage |
|  |  | (c) Consider use of a flow diagram |  |  |
| Descriptive data | 14* | (a) Give characteristics of study participants (eg demographic, clinical, social) and information on exposures and potential confounders | 8 | Sample collection and storage |
|  |  | (b) Indicate number of participants with missing data for each variable of interest | - | There was no missing data in this study. |
|  |  | (c) *Cohort study*—Summarise follow-up time (eg, average and total amount) | - | - |
| Outcome data | 15* | *Cohort study*—Report numbers of outcome events or summary measures over time | *-* | *-* |
|  |  | *Case-control study—*Report numbers in each exposure category, or summary measures of exposure | 21 | Figure4 |
|  |  | *Cross-sectional study—*Report numbers of outcome events or summary measures | *-* | *-* |
| Main results | 16 | (*a*) Give unadjusted estimates and, if applicable, confounder-adjusted estimates and their precision (eg, 95% confidence interval). Make clear which confounders were adjusted for and why they were included | 21 | Figure4 |
|  |  | (*b*) Report category boundaries when continuous variables were categorized | 12, 13 | Quantification of microbial effects on cell viability |
|  |  | (*c*) If relevant, consider translating estimates of relative risk into absolute risk for a meaningful time period | 13 | Quantification of microbial effects on cell viability (We also calculated Population Attributable Risk Percent (PARP) by integrating the relative risk values of specific bacteria with their prevalence in the tissue.) |

Continued on next page

| Other analyses | 17 | Report other analyses done—eg analyses of subgroups and interactions, and sensitivity analyses | 14, 17, 19, 24 | Figure1, Figure2, Figure3, Figure5 |
| --- | --- | --- | --- | --- |
| Discussion | | | | |
| Key results | 18 | Summarise key results with reference to study objectives | 26 | we developed a novel spatial host-microbiome profiling approach that, to the best of our knowledge, is the first to enable simultaneous species-level identification of bacteria and host transcriptomics. Using this novel approach, we demonstrated increased bacterial translocation in CD, with a significant association between the extent of translocation and disease prognosis, while also revealing distinct host transcriptome alterations in response to translocation of various bacterial species. Furthermore, we identified and characterized potentially beneficial and pathogenic microbiomes associated with CD, including several newly discovered risk-modulating bacterial species. |
| Limitations | 19 | Discuss limitations of the study, taking into account sources of potential bias or imprecision. Discuss both direction and magnitude of any potential bias | 30 | This study has several limitations. First, the limited number of individuals in our study may introduce potential biases, although the total number of cells analyzed was substantial (13,876). Future studies should include more samples from diverse ethnic backgrounds to improve the generalizability of the findings. Second, the spatial transcriptomic technology used in our study lacks single-cell resolution, resulting in the mixing of various cell types within spots. Although we employed cell2location to classify areas based on cell combinations, heterogeneity within areas of the same type may still exist, meaning that there could be slight differences in cellular composition among areas classified as the same type. This heterogeneity could potentially influence the comparisons made within the same type of area, such as differences of gene expression in immune cell-rich area due to bacterial exposure. However, the emergence of high-resolution single-cell spatial transcriptomics techniques offers opportunities to apply our algorithm to more refined spatial data in future investigations, which could help reduce the impact of cellular heterogeneity on our analyses. |
| Interpretation | 20 | Give a cautious overall interpretation of results considering objectives, limitations, multiplicity of analyses, results from similar studies, and other relevant evidence | 30, 31 | In conclusion, our study introduces a novel spatial host-microbiome profiling approach that enables the simultaneous profiling of the host transcriptome and bacterial species at a high taxonomic resolution in the ileal tissues of pediatric CD patients. This approach allowed us to identify increased bacterial translocation in CD tissues, as well as the potential prognostic value of assessing bacterial infiltration in intestinal tissues. We also discovered specific beneficial and pathogenic microbiomes associated with CD pathogenesis and suggested potential mechanism by which these microbes may influence disease progression, such as the modulation of host cell apoptosis pathways. The identification of several newly discovered beneficial microbiomes provides promising candidates for the development of novel microbiome-based therapeutics for CD. Our spatial host-microbiome sequencing approach offers a valuable method for understanding the intricate interactions between gut microbes and host cells in the context of CD pathogenesis. |
| Generalisability | 21 | Discuss the generalisability (external validity) of the study results | 27, 28 |  |
| Other information | |  | | |
| Funding | 22 | Give the source of funding and the role of the funders for the present study and, if applicable, for the original study on which the present article is based | 32 | This study was supported by a grant from the MD-PhD Physician-Scientist Training Program from the Korea Health Industry Development Institute (KHIDI), Ministry of Health and Welfare of the Republic of Korea. |

*Give information separately for cases and controls in case-control studies and, if applicable, for exposed and unexposed groups in cohort and cross-sectional studies.

**Note:** An Explanation and Elaboration article discusses each checklist item and gives methodological background and published examples of transparent reporting. The STROBE checklist is best used in conjunction with this article (freely available on the Web sites of PLoS Medicine at http://www.plosmedicine.org/, Annals of Internal Medicine at http://www.annals.org/, and Epidemiology at http://www.epidem.com/). Information on the STROBE Initiative is available at www.strobe-statement.org.
