# Supplementary material for: Spatial host-microbiome profiling demonstrates bacterial-associated host transcriptional alterations in pediatric ileal Crohn’s disease
Source: Microbiome. 2025 Aug 23;13:189. doi: 10.1186/s40168-025-02178-8 (PMC12374449; doi:10.1186/s40168-025-02178-8)

**Supplementary Methods**

**Bacterial-cell type co-localization analysis**

Bacterial-cell type co-localization probabilities were estimated using cell type fraction data generated by Cell2location analysis. For each spatial spot, we calculated co-localization probabilities by multiplying the estimated fraction of each cell type with the bacterial read counts detected in that spot. These values were then summed across all spots within each sample to generate a co-localization probability matrix. The resulting matrix was visualized as a heatmap (Supplementary Figure 5), where rows represent bacterial species and columns represent the 42 different cell types identified through Cell2location deconvolution. Higher values in the heatmap indicate stronger co-localization between specific bacteria and cell types. This method allows quantitative estimation of which cell types are most frequently associated with beneficial or pathogenic microbiomes in spatial transcriptomics data.

**Supplementary Figures**

**Supplementary Figure 1** Comparison of spatial versus bulk sequencing detection and single versus double human read removal for gut-residing and false positive bacterial species (A, B) Comparison between spatial sequencing and bulk shotgun sequencing detection for genuine gut-residing species (Faecalibacterium prausnitzii and Escherichia coli) and potential false positive species (Guillardia theta and Puccinia striiformis). (C, D) Comparison of bacterial read counts between single (Bowtie2 only) and double (Bowtie2 + BWA) human read removal processes for genuine gut-residing species (Faecalibacterium prausnitzii and Escherichia coli) and potential false positive species (Guillardia theta and Puccinia striiformis).

**
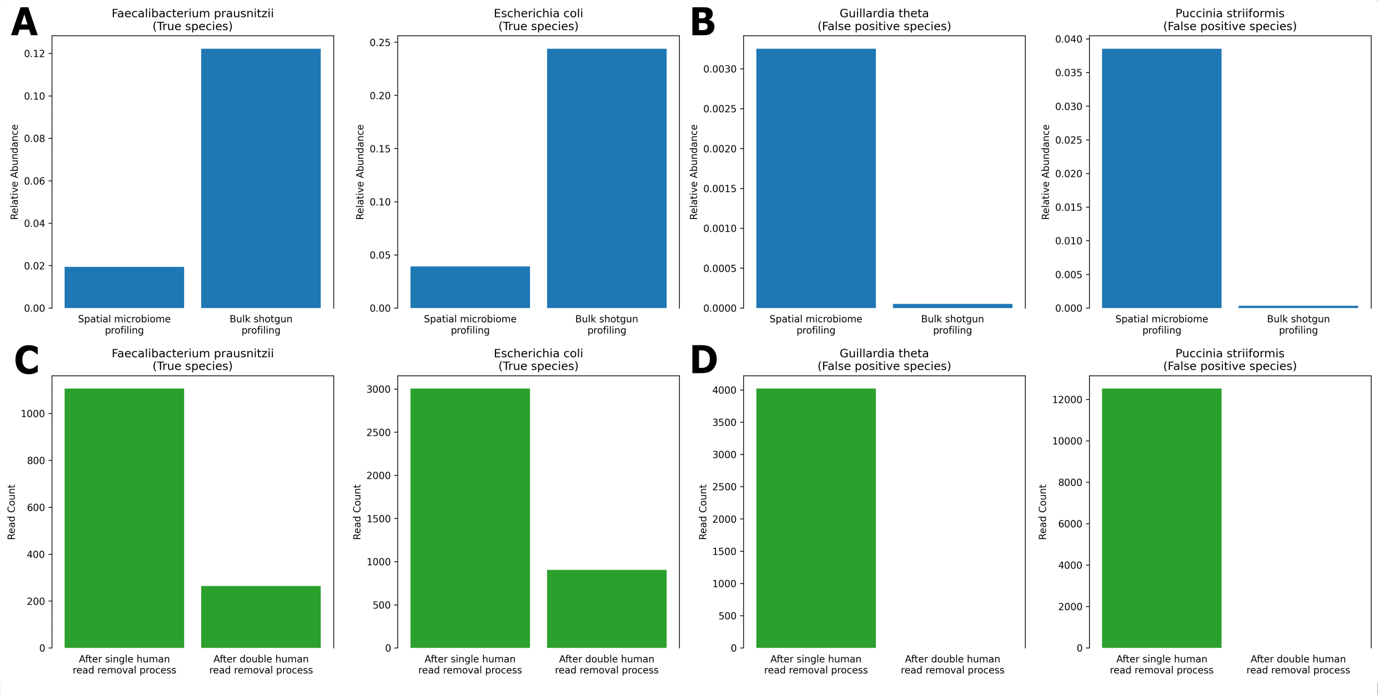
**

**Supplementary Figure 2** Validation of spatial microbiome profiling by Gram staining of adjacent tissue sections. (A) Spatial distribution of bacterial reads (red intensity) across tissue sections from CD patients and controls, as detected by spatial host-microbiome profiling. (B) Histological appearance (H&E staining) of corresponding tissue sections showing tissue architecture. (C) Gram staining of adjacent tissue sections demonstrating bacterial localization patterns with Gram-positive bacteria appearing as dark purple clusters.


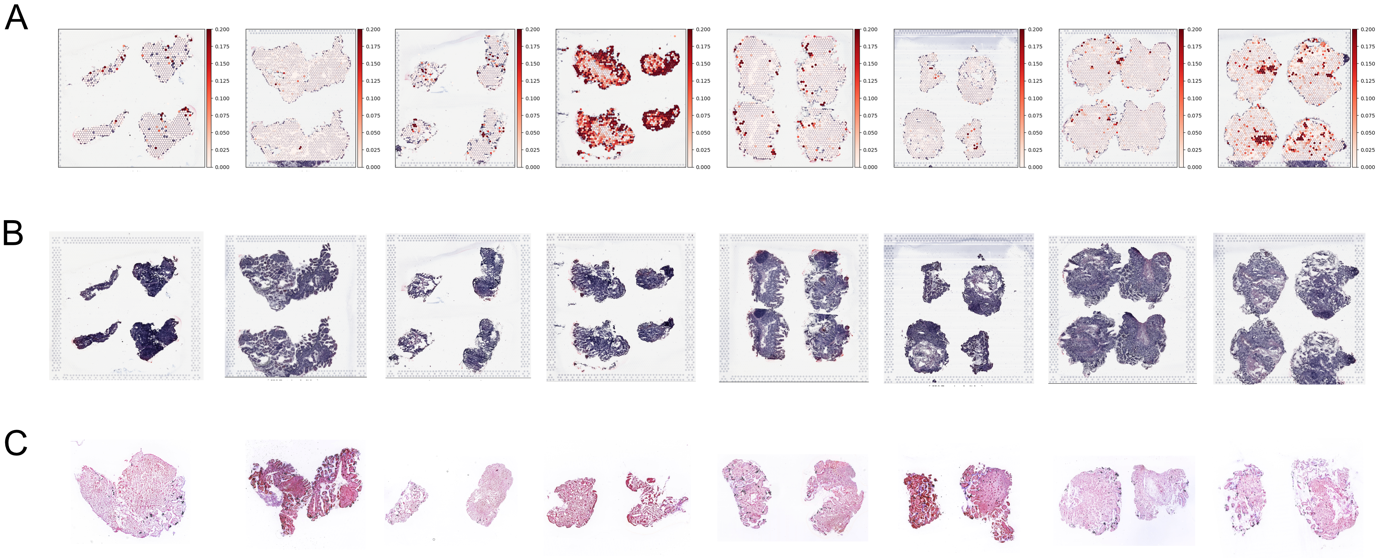


**Supplementary Figure 3** High-magnification correlation between spatial microbiome sequencing and Gram staining. (A-C) Higher magnification views of selected regions showing correlation between spatial sequencing bacterial signals and Gram-positive bacterial clusters (dark purple) within tissue structures. The spatial distribution patterns of bacteria detected by sequencing show overall similarity with bacterial localization observed in Gram-stained adjacent sections, validating the spatial microbiome profiling approach.


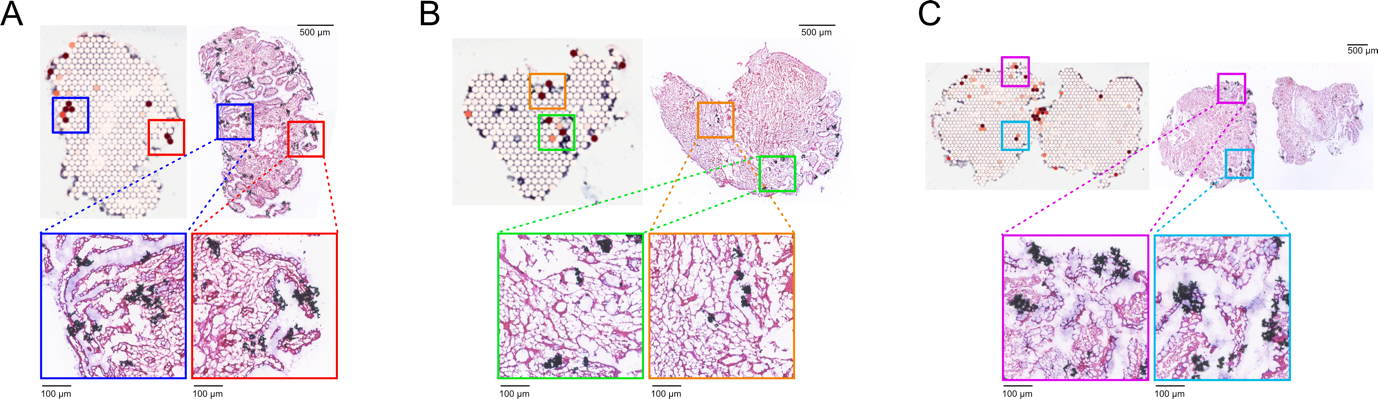


**Supplementary Figure 4** Population Attributable Risk Percent (PARP) analysis of bacterial species in pediatric Crohn's disease. PARP values for bacterial species, calculated by integrating relative risk values with tissue prevalence. Green bars indicate beneficial bacteria that reduce intestinal barrier disruption, while red bars represent pathogenic bacteria that increase tissue damage.

**
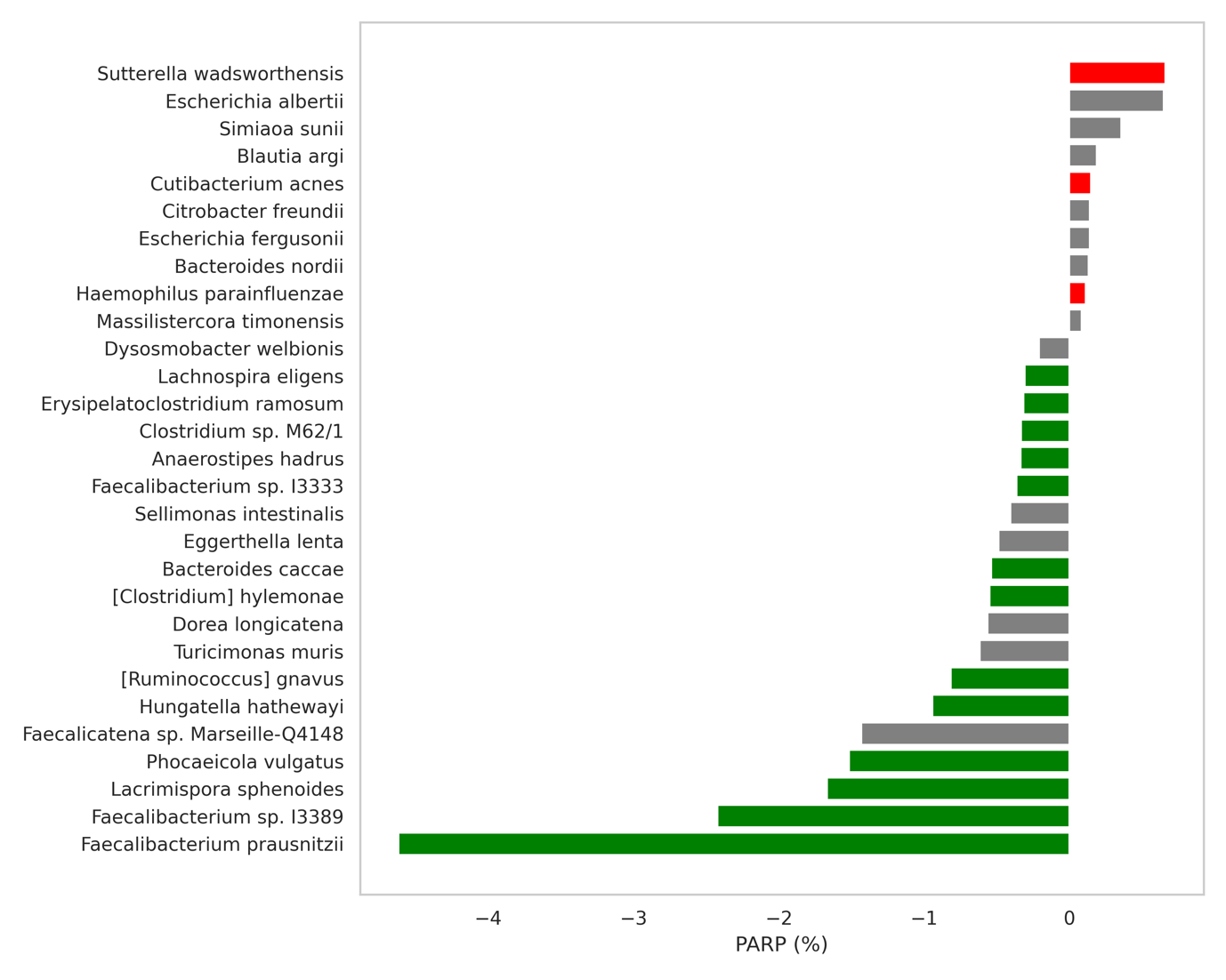
**

**Supplementary Figure 5** Cell type-specific co-localization probabilities of bacterial species in pediatric ileal Crohn's disease tissues. (A) Heatmap showing co-localization probabilities between bacterial species and immune cell types. Color intensity represents the strength of co-localization probability calculated by multiplying Cell2location-derived cell type fractions with bacterial counts per spot. (B) Co-localization probabilities between bacterial species and non-immune cell types. (C) Log2 fold change comparison of co-localization probabilities between beneficial and pathogenic microbiomes across different cell types. Green bars indicate cell types with higher co-localization probability for beneficial microbiomes, while red bars indicate higher probability for pathogenic microbiomes. Statistical significance is indicated by asterisks (*P < 0.05, **P < 0.01, ***P < 0.001).


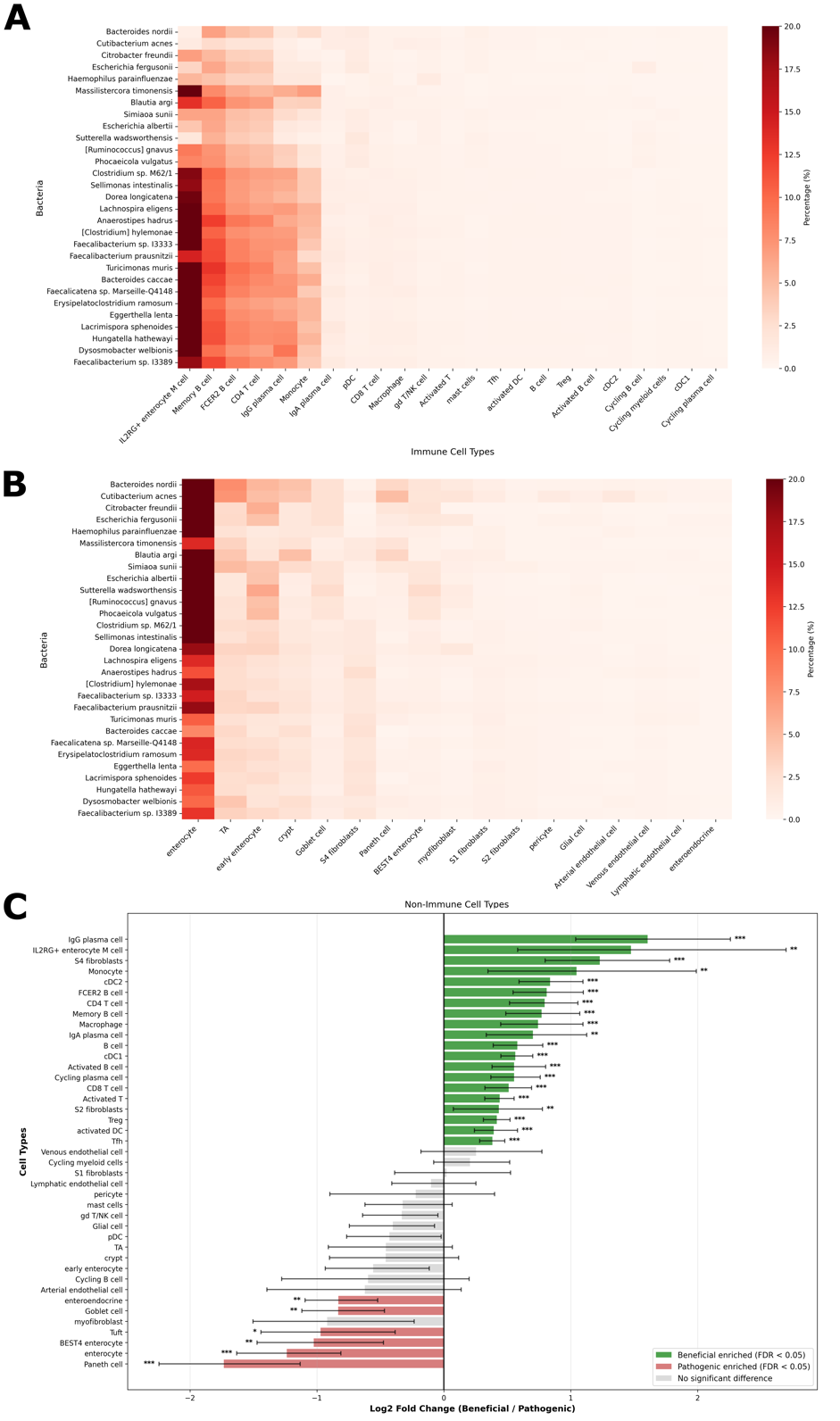


**Supplementary Figure 6** Strain-level relative risk analysis of bacterial species associated with reduced cell viability in pediatric Crohn's disease. Relative risk (RR) values for strain-level bacterial species and their association with reduced cell viability in CD tissues. Red circles indicate pathogenic strains with RR > 1.0, while gray circles reƒpresent beneficial strains with RR < 1.0. Error bars represent 95% confidence intervals. Only statistically significant results after Benjamini-Hochberg correction are shown.


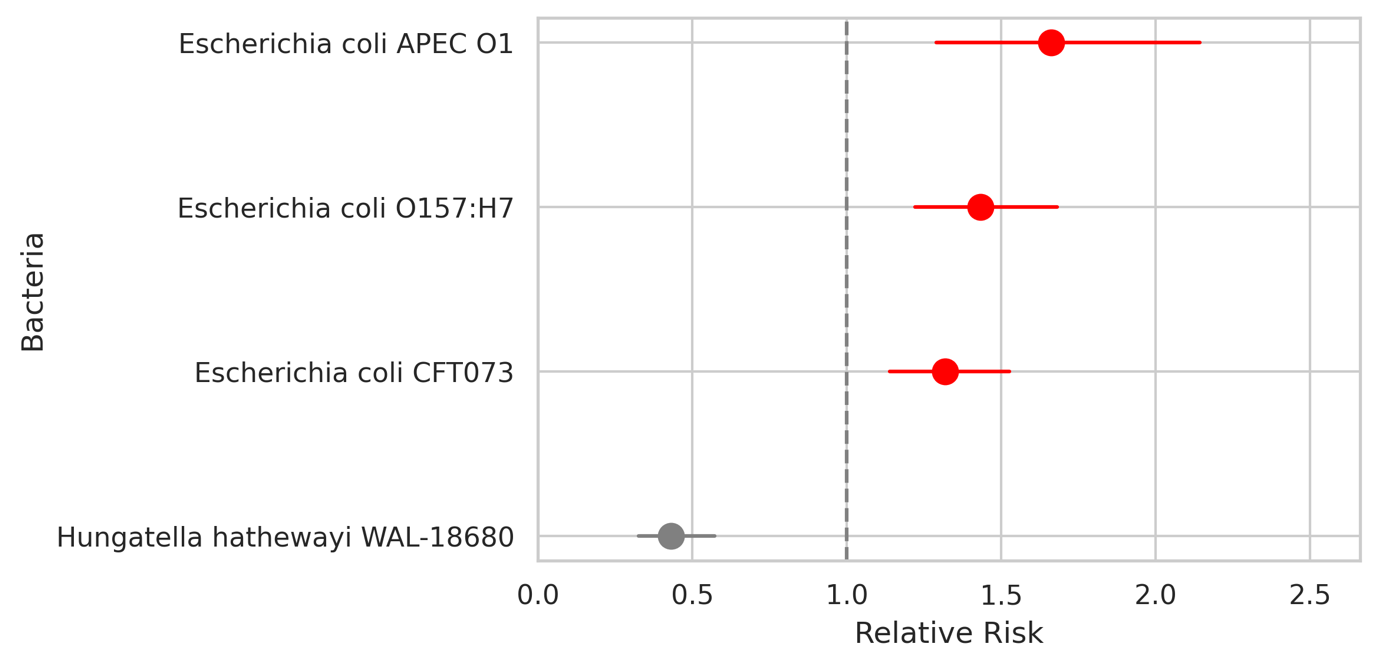


**Supplementary Figure 7** Gene contribution to Principal Component 1 in bacterial presence correlation analysis. Bar chart displaying the top 20 genes with the highest absolute contribution to Principal Component 1 (PC1) in the correlation analysis between bacterial presence and host gene expression levels.


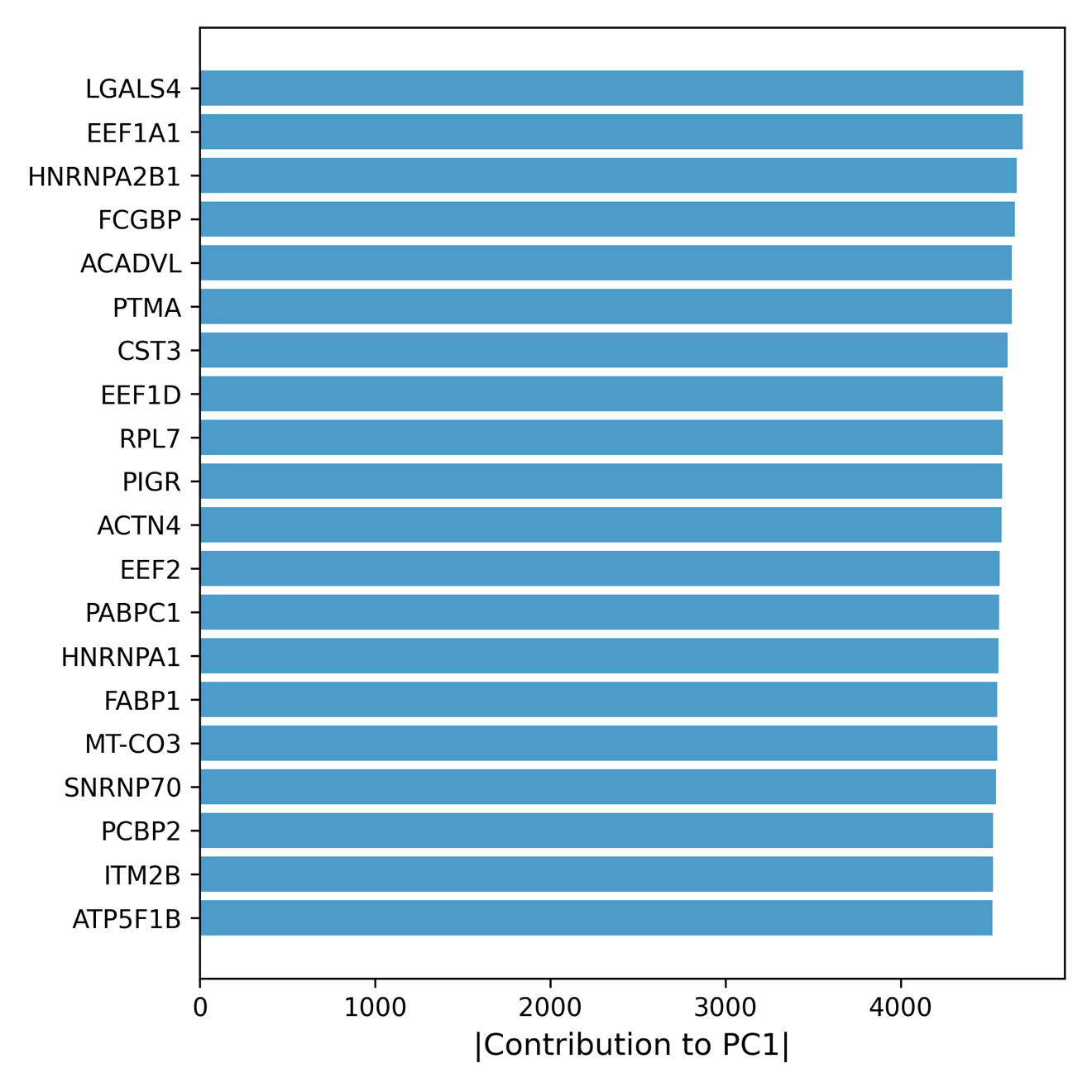

Supplement: Supplementary file 3 — Additional file 2: Supplementary methods. Supplementary Figure S1 Comparison of spatial versus bulk sequencing detection and single versus double human read removal for gut-residing and false positive bacterial species (A, B) Comparison between spatial sequencing and bulk shotgun sequencing detection for genuine gut-residing species (Faecalibacterium prausnitzii and Escherichia coli) and potential false positive species (Guillardia theta and Puccinia striiformis). (C, D) Comparison of bacterial read counts between single (Bowtie2 only) and double (Bowtie2 + BWA) human read removal processes for genuine gut-residing species (Faecalibacterium prausnitzii and Escherichia coli) and potential false positive species (Guillardia theta and Puccinia striiformis). Supplementary Figure S2 Validation of spatial microbiome profiling by Gram staining of adjacent tissue sections. (A) Spatial distribution of bacterial reads (red intensity) across tissue sections from CD patients and controls, as detected by spatial host-microbiome profiling. (B) Histological appearance (H&E staining) of corresponding tissue sections showing tissue architecture. (C) Gram staining of adjacent tissue sections demonstrating bacterial localization patterns with Gram-positive bacteria appearing as dark purple clusters. Supplementary Figure S3 High-magnification correlation between spatial microbiome sequencing and Gram staining. (A-C) Higher magnification views of selected regions showing correlation between spatial sequencing bacterial signals and Gram-positive bacterial clusters (dark purple) within tissue structures. The spatial distribution patterns of bacteria detected by sequencing show overall similarity with bacterial localization observed in Gram-stained adjacent sections, validating the spatial microbiome profiling approach. Supplementary Figure S4 Population Attributable Risk Percent (PARP) analysis of bacterial species in pediatric Crohn's disease. PARP values for bacterial species, calculate [file 40168_2025_2178_MOESM2_ESM.docx]
